# Ghrelin Gene Deletion Alters Pulsatile Growth Hormone Secretion in Adult Female Mice

**DOI:** 10.3389/fendo.2021.754522

**Published:** 2021-10-13

**Authors:** Rim Hassouna, Gimena Fernandez, Nicolas Lebrun, Oriane Fiquet, Ferdinand Roelfsema, Alexandra Labarthe, Philippe Zizzari, Catherine Tomasetto, Jacques Epelbaum, Odile Viltart, Christophe Chauveau, Mario Perello, Virginie Tolle

**Affiliations:** ^1^ Université de Paris, UMR-S 1266 INSERM, Institute of Psychiatry and Neuroscience of Paris, Paris, France; ^2^ Laboratory of Neurophysiology of the Multidisciplinary Institute of Cell Biology [IMBICE, Argentine Research Council (CONICET) and Scientific Research Commission, Province of Buenos Aires (CIC-PBA). National University of La Plata], La Plata, Buenos Aires, Argentina; ^3^ Department of Internal Medicine, Section of Endocrinology and Metabolism, Leiden University Medical Center, Leiden, Netherlands; ^4^ Institut de génétique et de biologie moléculaire et cellulaire (IGBMC), UMR7104 CNRS/U1258 INSERM, Université de Strasbourg, Illkirch, France; ^5^ UMR CNRS/MNHN 7179, Mécanismes Adaptatifs et Evolution, Brunoy, France; ^6^ Université de Lille, Faculté des Sciences et Technologies, Villeneuve d’Ascq, France; ^7^ Marrow Adiposity and Bone Lab - MABLab ULR 4490, Univ. Littoral Côte d’Opale, Boulogne-sur-Mer, Univ. Lille and CHU Lille, Lille, France

**Keywords:** ghrelin, growth hormone, food intake, sexual dimorphism, pulsatility

## Abstract

Using preproghrelin-deficient mice (*Ghrl-/-*), we previously observed that preproghrelin modulates pulsatile growth hormone (GH) secretion in post-pubertal male mice. However, the role of ghrelin and its derived peptides in the regulation of growth parameters or feeding in females is unknown. We measured pulsatile GH secretion, growth, metabolic parameters and feeding behavior in adult *Ghrl-/-* and *Ghrl+/+* male and female mice. We also assessed GH release from pituitary explants and hypothalamic growth hormone-releasing hormone (GHRH) expression and immunoreactivity. Body weight and body fat mass, linear growth, spontaneous food intake and food intake following a 48-h fast, GH pituitary contents and GH release from pituitary explants *ex vivo*, fasting glucose and glucose tolerance were not different among adult *Ghrl-/-* and *Ghrl+/+* male or female mice. *In vivo*, pulsatile GH secretion was decreased, while approximate entropy, that quantified orderliness of secretion, was increased in adult *Ghrl-/-* females only, defining more irregular GH pattern. The number of neurons immunoreactive for GHRH visualized in the hypothalamic arcuate nucleus was increased in adult *Ghrl-/-* females, as compared to *Ghrl+/+* females, whereas the expression of GHRH was not different amongst groups. Thus, these results point to sex-specific effects of preproghrelin gene deletion on pulsatile GH secretion, but not feeding, growth or metabolic parameters, in adult mice.

## Introduction

The ghrelin gene (GHRL) encodes proghrelin prohormone, which in turn gives rise to acylated and non-acylated ghrelin as well as obestatin, which are mainly secreted from cells of the stomach (1). The most studied proghrelin-derived product is the 28-residue acylated peptide ghrelin that acts *via* the growth hormone secretagogue receptor (GHSR). Ghrelin administration in humans and rodents induces a plethora of effects (2, 3) including stimulation of GH secretion (2, 4). In rodents, this effect is principally mediated through GHRH neurons of the hypothalamic arcuate nucleus (ArcN) (5). Also, ghrelin treatment rapidly increases food intake (6), glycaemia (7–9), glucocorticoid secretion (10) and affects cognitive behaviors (11). Strikingly, the notion that endogenous ghrelin plays a leading physiological role has been challenged by studies showing that genetically-modified mice lacking ghrelin display minor alterations (12). In particular, adult *Ghrl-/-* mice fed on a regular diet show food intake, body weight, body size and body composition indistinguishable from those observed in wild-type littermates (13–17). Also, mice lacking the enzyme ghrelin-O-acyl-transferase (GOAT), which n-acetylates ghrelin, and mice with ablation of proghrelin-expressing cells show no food intake, body growth nor body weight alterations (18–22). Despite that ghrelin system is believed to play a major role under energy deficit conditions, such as fasting, when plasma ghrelin levels increase, fasted *Ghrl-/-* mice show no alterations in plasma parameters (e.g., glucose, insulin, leptin) nor in compensatory hyperphagia when refed (23–25). Still, young *Ghrl-/-* male mice show reduced amplitude of GH secretory pulses (26). Moreover, some studies found that *Ghrl-/-* mice fed on a high-fat diet (HFD) show lower body weight and fat mass (24, 27, 28). Interestingly, GHSR-deficient (*Ghsr-/-*) mice usually show more robust alterations, as compared to *Ghrl-/-* mice, which has been attributed to the fact that the latter retain ligand-independent effects of GHSR and/or signaling of other proghrelin-derived peptides (25, 29).

The vast majority of the research investigating *Ghrl-/-* mice has been conducted in males. Specifically, some of the above referred studies did not include females at all (24, 25, 30). Other studies merely assessed body weight or food intake whereas the assessment of plasma hormone levels was exclusively performed in males (17, 23). Notably, several studies suggest a modulatory role of sex on ghrelin actions (31–34), but sex differences in *Ghrl-/-* mice, and specifically on GH secretion, have not been investigated thoroughly. In GHSR-deficient mice, only females fed on standard chow diet had lower body weight and adiposity, as compared to wild-type littermates (35). Furthermore, *Ghsr-/-* female but not male mice fed on a HFD exhibited reduced taste responsiveness to linoleic acid compared to wild-type littermates (36). Finally, adult *Ghsr-/-* female but not male mice had reduced pulsatile GH secretion (37). We thus questioned a possible role of ghrelin gene in regulating GH secretion and its physiological consequences in female mice during adulthood, when the changes in pulsatile GH secretion and pattern associated with the rapid linear growth and pubertal maturation has ended (38, 39). Specifically, we investigated if ghrelin gene deletion induces sex-specific dimorphic effects on GH secretion, feeding behavior or body and metabolic parameters in adult 20-40-week-old mice.

## Materials and Methods

### Animals

*Ghrl-/-* mice were originally obtained from Dr Tomasetto (IGBMC, France) and backcrossed on a C57BL/6J genetic background (26). Heterozygous mice were raised at the Institute of Psychiatry and Neuroscience of Paris (INSERM UMR-S 1266) and bred to obtain *Ghrl*-/- mice and wild-type littermates (*Ghrl*+/+ mice). Offspring were genotyped as previously described (26) and housed in a room under controlled illumination (7:00 to 19:00) and temperature (22–24°C). Mice had free access to chow diet (3% fat, 16% protein, 60% carbohydrate, 4% fibers, 2.79 kcal/g, Safe A04, France) and water, except when indicated. Experiments were conducted in 20-40-week-old male and random cycling female mice.

### Assessment of Body Weight, Body Length and Body Composition

A cohort of anesthetized mice (6 *Ghrl+/+* and 7 *Ghrl-/-* males*;* 8 *Ghrl+/+* and 7 *Ghrl-/-* females) was used to assess naso-anal distance. Another cohort of mice (4-7 *Ghrl+/+* and 4-6 *Ghrl-/-* males*;* 4-9 *Ghrl+/+* and 4-6 *Ghrl-/-* females) was dissected to weigh pituitary gland, liver, heart, spleen, pancreas, testis/ovaries, kidneys, adrenals and fat depots (mesenteric, inguinal, perirenal, perigonadal, interscapular brown adipose tissue).

### Repeated Blood Sampling for GH Assay

Ultradian GH secretion was assessed as previously described (26, 40). Briefly, mice (5 *Ghrl+/+* and 8 *Ghrl-/-* males*;* 8 *Ghrl+/+* and 6 *Ghrl-/-* females) were acclimated to handling and blood sampling collection to minimize stress. Then, sequential tail-tip whole venous blood samples (2 μl/sample) were collected every 10-min over a 6-h-sampling period (9:00 to 15:00) (See **Supplementary File** for details).

### Assessment of Pulsatile GH Secretion

GH concentration time series were analyzed using an automated deconvolution method following established parameters (41). Measures include the number of pulses (over the 6-h sampling period) and mean pulse mass (*i.e.*, mean of the summed pulses) as well as basal (*i.e.*, non-pulsatile), pulsatile (*i.e.*, sum of individual GH pulses) and total GH secretion (*i.e.*, sum of basal plus pulsatile). The orderliness of GH secretion was calculated by Jack-Knife Approximate Entropy (JkApEn) as described earlier (42, 43) (See **Supplementary File** for details).

### GH Pituitary Content and GH Release From Pituitary Explants

Two different cohorts of mice were used to assess total GH pituitary contents (12 *Ghrl+/+* and 7 *Ghrl-/-* males*;* 5 *Ghrl+/+* and 5 *Ghrl-/-* females) and basal and GHRH-stimulated GH release from perifused pituitary explants (4 *Ghrl+/+* and 4 *Ghrl-/-* males*;* 4 *Ghrl+/+* and 4 *Ghrl-/-* females) as previously described (26) (See **Supplementary File** for details). Samples were frozen at -20°C until GH determination.

### GH Enzyme Immuno-Assay

GH concentrations in whole blood, pituitary contents and media were determined using an in-house mouse GH ELISA (40). (See **Supplementary File** for details). The assay sensitivity was 0.038 ng/mL, and intra- and inter-assay coefficients of variations were 3.2% and less than 8.75%, respectively.

### Assessment of Spontaneous Food Intake

Mice (8 *Ghrl+/+* and 6 *Ghrl-/-* males*;* 7 *Ghrl+/+* and 8 *Ghrl-/-* females) were individually housed in automated feeding stations equipped with high precisions sensors (LabMaster System, TSE Systems, Germany) to record spontaneous food intake and meal patterns as previously reported (44) (See **Supplementary File** for details).

### Fasting-Refeeding Protocol

As described before (25), individually housed mice (5 *Ghrl+/+* and 5 *Ghrl-/-* males*;* 7 *Ghrl+/+* and 6 *Ghrl-/-* females) were fasted at 10:00 and refed 48-h later. Body weight and food intake was daily monitored at 10:00 for four days after refeeding. Food intake was calculated by subtracting the weight of the remaining food at 10:00 to the weight of the initial food. Importantly, mice fully tolerated a 48-h fasting period (25, 45).

### Assessment of Blood Glucose and Glucose Tolerance Test

Blood glucose was measured with a glucometer (GlucoFix Premium, Menarini Diagnostics) at 10:00 and at 18:00 in fed conditions as well as at 10:00 following a 24-h fast (8 *Ghrl+/+* and 6 *Ghrl-/-* males; 8 *Ghrl+/+* and 8 *Ghrl-/-* females). After fasting, glucose was measured before and 15-, 30-, 60- and 120-min after glucose intraperitoneal injection (2 g/kg body weight).

### GHRH Immunostaining and Quantification

Brains of perfused mice (3 *Ghrl+/+* and 5 *Ghrl-/-* males; 5 *Ghrl+/+* and 8 *Ghrl-/-* females) were post-fixed, frozen and coronally cut at 40 µm into four equal series. Chromogenic immunohistochemistry against GHRH was performed as described before (46), using a previously validated rabbit anti-GHRH antibody (47) (immunoserum L0851, 1:10000) for 48-h at 4°C. Then, sections were sequentially incubated with a biotinylated anti-rabbit antibody and the avidin-biotin-peroxidase complex. A visible black/purple signal was developed with a diaminobenzidine/nickel solution (See **Supplementary File** for details). Quantifications were performed in the ArcN between bregma -1.58 and -2.06 mm, using the anatomical limits, according to the Paxinos mouse brain atlas (48). Total GHRH-immunoreactive cells were quantified, and data were expressed as positive cells (GHRH+) per section. Blind quantitative analysis was performed independently by two observers.

### Real-Time Quantitative PCR Measurement

Hypothalami were quickly dissected, frozen in liquid nitrogen and stored at -80°C. The mRNAs were extracted with Trizol reagent and cDNAs were obtained from the reverse transcription of total RNA (ThermoFisher, Waltham, MA, USA). The mRNA levels of *Ghrh* and *Ghsr* were quantified relative to the housekeeping genes *Ppia* and *Gapdh*. Relative quantification (RQ) was calculated relative to the *Ghrl*+/+ males (See **Supplementary File** for details).

### Statistical Analysis

Data are expressed as mean ± SEM, and statistical analyses were performed using Statview software (SAS institute) or GraphPad Prism (GraphPad Software). Differences across sex and genotype were identified by 2-way ANOVA, followed by multiple comparisons, using Bonferroni *post-hoc* analysis. Differences were considered significant when p<0.05.

## Results

### Body Weight, Organ Weights, Body Fat Partitioning, Metabolic Parameters, Locomotor Activity, Spontaneous Food Intake and Fasting-Induced Food Intake Are Unaltered in Adult *Ghrl-/-* Mice

**Figure 1:**
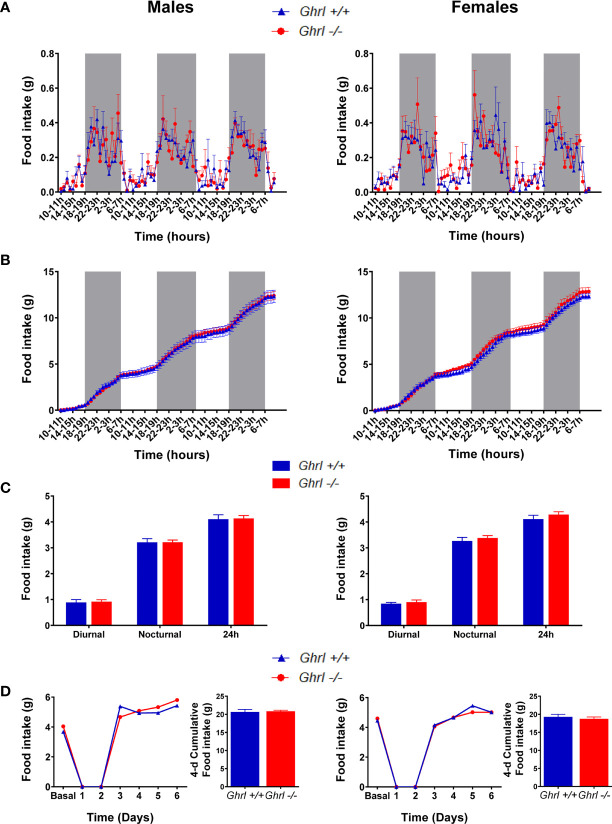
Spontaneous food intake and food intake in a fast-refeeding protocol in male and female *Ghrl-/-* mice. Feeding parameters in adult male and female *Ghrl+/+* and *Ghrl-/-* mice showing spontaneous food intake **(A)** and cumulative food intake **(B)** measured every hour over 3 days as well as diurnal, nocturnal and 24-h food intake averaged over 3 days **(C)**. Daily food intake and 4-days cumulative food intake during re-feeding **(D)** in mice fasted for 48-h and then allowed free access to food. Light and dark phases are denoted by white and grey rectangles on the x-axis. Data represent mean ± SEM. Number of mice in panels **(A–C)** males (n=8 *Ghrl+/+* males and 7 *Ghrl-/-*), females (n=8 *Ghrl+/+* and 8 *Ghrl-/-*). Number of mice in panel D: males (n=5 *Ghrl+/+* males and 5 *Ghrl-/-*), females (n=7 *Ghrl+/+* and 6 *Ghrl-/-*).

As shown in **Table 1**, body weight, naso-anal distance and organ weights were not different in adult *Ghrl-/-* as compared to *Ghrl+/+* males and females, respectively. Moreover, fed morning, fed evening and 24-h fasted blood glucose levels as well as glucose tolerance were not different in adult *Ghrl-/-* as compared to *Ghrl+/+* males and females. Spontaneous diurnal and nocturnal food intake, fasting-induced food intake (**Figure 1**) and home cage ambulatory activity (not shown) were not different in adult *Ghrl-/-* as compared to *Ghrl+/+* males and females. In addition, meal number, mean meal size and mean meal duration in *ad libitum* fed mice were not different amongst genotypes (not shown). Finally, parameters of bones architecture measured by micro-CT were not different in adult *Ghrl-/-* as compared to *Ghrl+/+* males and females (not shown), although a tendency toward reduction of bone volume fraction was observed (volume of mineral bone per unit volume of the sample, BV/TV; ANOVA, genotype effect: p=0.0894, sex effect: p<0.0001, sex x genotype effect: p=0.9014) in 80-week-old mice only.

**Table 1:** Growth and body parameters, body fat partitioning and metabolic parameters in male and female *Ghrl-/-* mice.

	Males		Females		Anova		
**Growth parameters**	***Ghrl+/+* (n=6-12)**	***Ghrl-/-* (n=7-15)**	***Ghrl+/+* (n=5-8)**	***Ghrl-/-* (n=5-7)**	**Sex**	**Genotype**	**Sex x Genotype**
Naso-anal distance (cm)	9.33 ± 0.08	10.19 ± 0.10	8.80 ± 0.22	8.93 ± 0.06	P=0.0608	p=0.0952	p=0.6642
GH (μg/μg proteins)	0.225 ± 0.021	0.278 ± 0.048	0.246 ± 0.045	0.245 ± 0.060	P=0.8819	p=0.5364	p=0.5129
**Body parameters and fat partitioning**	***Ghrl+/+* (n=4-7)**	***Ghrl-/-* (n=4-6)**	***Ghrl+/+* (n=4-9)**	***Ghrl-/-* (n=4-6)**	**Sex**	**Genotype**	**Sex x Genotype**
Body weight (g)	33.1 ± 2.0	33.9 ± 1.4	25.2 ± 0.9	23.8 ± 0.7	P<0.0001	p=0.8415	p=0.4342
Pituitary gland (mg)	1.3 ± 0.3	0.9 ± 0.1	1.7 ± 0.2	2.5 ± 1.3	P=0.0994	p=0.7151	p=0.3061
Liver (mg)	1303.4 ± 80.3	1264.0 ± 93.3	1090.9 ± 83.8	996.5 ± 31.2	P=0.0066	p=0.4157	p=0.7367
Heart (mg)	167.9 ± 6.4	166.7 ± 8.1	130.8 ± 4.2	130.5 ± 5.6	P<0.0001	p=0.9043	p=0.9404
Spleen (mg)	97.8 ± 7.8	95.2 ± 5.1	82.6 ± 7.5	79.7 ± 4.9	P=0.0403	p=0.7005	p=0.9835
Pancreas (mg)	410.6 ± 29.5	388.2 ± 32.9	402.4 ± 43.0	359.5 ± 43.9	P=0.6486	p=0.4205	p=0.7989
Testis/ovary (mg)	98.8 ± 6.8	98.8 ± 4.2	78.0 ± 7.6	79.8 ± 3.8	–	p=0.8834	–
Kidneys (mg)	450.6 ± 27.3	401.8 ± 12.6	389.4 ± 42.2	295.9 ± 17.5	P=0.0169	p=0.0387	p=0.4972
Adrenals (mg)	4.1 ± 0.5	3.2 ± 0.4	6.4 ± 0.4	6.3 ± 0.3	P<0.001	p=0.2499	p=0.3760
Mesenteric AT (mg)	420.7 ± 49.9	529.0 ± 88.7	286.8 ± 51.2	184.7 ± 44.5	P=0.0007	p=0.9597	p=0.0977
Inguinal AT (mg)	85.6 ± 17.0	68.5 ± 11.7	40.2 ± 5.4	34.2 ± 3.5	P=0.001	p=0.2908	p=0.6116
Perigonadal AT (mg)	815.9 ± 236.8	1137.2 ± 236.3	522.7 ± 41.0	446 ± 74.9	–	p=0.4591	–
Perirenal AT (mg)	446.4 ± 125.4	502.3 ± 79.7	186.4 ± 16.3	163.8 ± 13.7	P=0.0004	p=0.8221	p=0.5967
Scapular Brown AT (mg)	211.6 ± 33.6	264.0 ± 38.4	140.1 ± 16.1	143.2 ± 20.7	P=0.0018	p=0.3224	p=0.3775
**Glucose parameters**	***Ghrl+/+* (n=8)**	***Ghrl-/-* (n=6)**	***Ghrl+/+* (n=8)**	***Ghrl-/-* (n=8)**	**Sex**	**Genotype**	**Sex x Genotype**
Fed morning glucose (mg/dL)	150 ± 6	138 ± 8	142 ± 4	139 ± 7	P=0.6350	p=0.2451	p=0.5073
Fed evening glucose (mg/dL)	138 ± 6	137 ± 4	134 ± 11	123 ± 13	P=0.3910	p=0.5751	p=0.6499
24h fasted glucose (mg/dL)	89 ± 3	86 ± 6	95 ± 4	94 ± 4	P=0.1070	p=0.5957	p=0.8293
AUC Glucose (GTT)	37543 ± 1862	34825 ± 3323	34592 ± 6525	30270 ± 2243	P=0.3657	p=0.3958	p=0.8455

Growth parameters, mass of organs (pituitary gland, liver, heart, spleen, pancreas, testis/ovary, kidney, adrenals), fat mass deposits (mesenteric, inguinal, perigonadal, perirenal and scapular brown adipose tissue), blood glucose parameters (Fed 10.00, Fed 18.00, 24h Fasted and AUC during glucose tolerance) were measured in adult Ghrl+/+ and Ghrl-/- male and female mice. Numbers of mice are indicated in parenthesis. Data represent mean± SEM. AT, Adipose Tissue; AUC, Area Under the Curve; GTT, Glucose Tolerance Test.

### The Ultradian Pattern of GH Secretion Is Altered in Adult *Ghrl-/-* Females, but Not in *Ghrl-/-* Males

**Figure 2:**
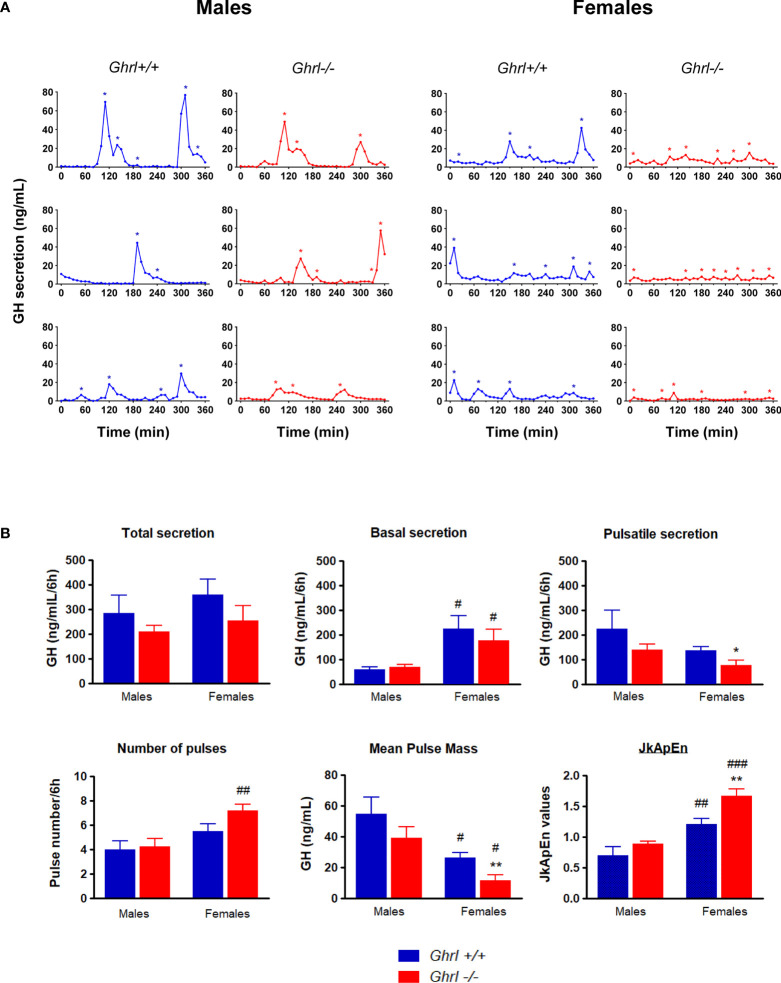
Analyses of ultradian GH secretion in male and female *Ghrl-/-* mice. Representative individual plasma GH secretory profiles in 36-week-old male and female *Ghrl+/+* and *Ghrl-/-* mice **(A)**. Deconvolution analyses and regularity parameters in adult *Ghrl+/+* and *Ghrl-/-* male and female mice showing total GH secretion, basal GH secretion, pulsatile GH secretion, number of pulses, mean pulse mass and JkApEn values **(B)**. Asterisks indicate the location of secretory peaks. Data represent mean ± SEM. Number of mice: males (n=5 *Ghrl+/+* males and 8 *Ghrl-/-*), females (n=8 *Ghrl+/+* and 6 *Ghrl-/-*). *p < 0.05, **p < 0.01 *Ghrl-/- versus Ghrl+/+* mice of the same sex. ^#^p < 0.05, ^##^p < 0.01, ^###^p < 0.0001 females *versus* males of the same genotype.

GH pituitary contents were not different in adult *Ghrl-/-*, as compared to *Ghrl+/+* males and females (**Table 1**). As reported before (37), we observed a sexually dimorphic GH secretion pattern. Indeed, mean pulse mass was higher in males than in females (p<0.05 in both *Ghrl+/+* and *Ghrl-/-* mice, *post-hoc* test), whereas basal GH secretion and JkApEn values were lower in males than in females (p<0.05 in both *Ghrl+/+* and *Ghrl-/-* mice for basal GH, p<0.01 in *Ghrl+/+* mice and p<0.0001 in *Ghrl-/-* mice for JkApEn, *post-hoc* test) (**Figure 2**). An increase in the number of GH pulses in females as compared to males was observed in *Ghrl-/-* mice only (p<0.01, *post-hoc* test). Furthermore, we observed that the regularity of GH secretion was decreased in *Ghrl-/-* female mice when compared to *Ghrl+/+* female mice (**Figure 2**). More precisely, pulsatile GH secretion and mean pulse mass were reduced by 43% and 56% respectively in *Ghrl-/-* females as compared to *Ghrl+/+* females (p<0.05 and p<0.01 for pulsatile GH and mean pulse mass, respectively, post-hoc test) while total and basal GH secretion were not significantly affected by ghrelin deletion. The maximal pulse amplitude was also reduced by 50% in *Ghrl-/-* compared to *Ghrl+/+* females (26 ± 4 versus 13 ± 3 ng/mL, p<0.05, *post-hoc* test). In contrast, JkApEn values increased by 27% in *Ghrl-/-* females as compared to *Ghrl+/+* females (p<0.01, *post-hoc* test). In males, we did not find genotype differences for any of the GH secretion parameters, however, the inter-individual variability of pulsatile GH, maximal pulse amplitude and mean pulse mass was higher than in females (see representative GH profiles).

### *In Vitro* Release of GH Is Unaltered in Pituitary Explants From Adult Male and Female *Ghrl-/-* and *Ghrl+/+* Mice

**Figure 3:**
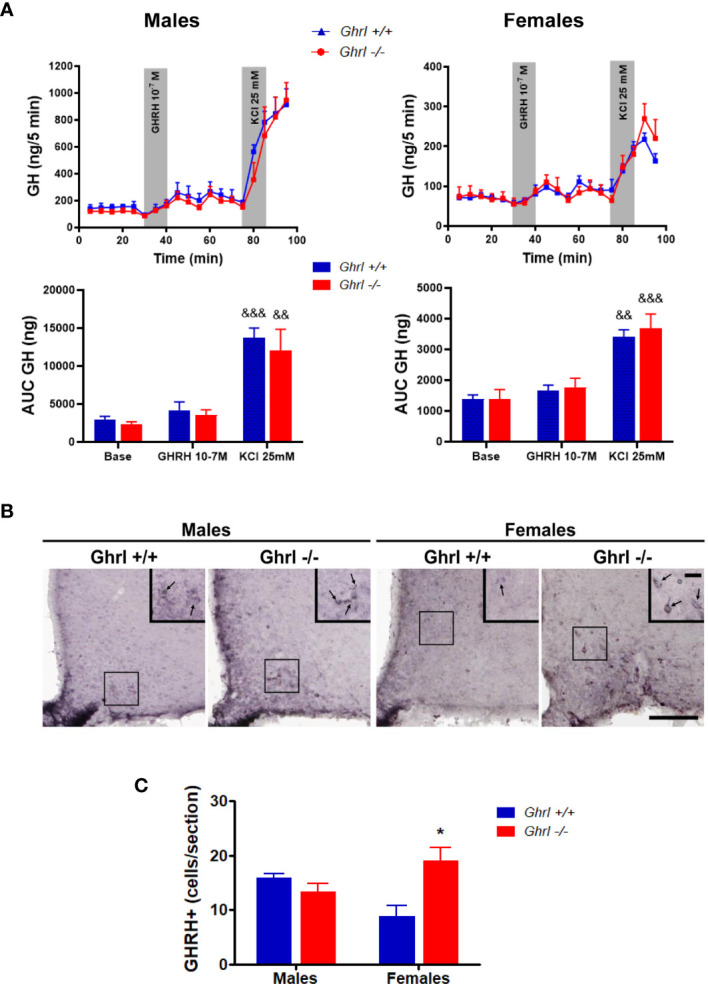
GH release from pituitary explants and GHRH immunoreactivity in the hypothalamus of male and female *Ghrl-/-* mice. Basal GH release, 10^-7^ M GHRH-induced GH release and 25 mM KCl induced GH release measured in adult male and female *Ghrl+/+* and *Ghrl-/-* mice. Note that the Y-axes scales are different for males and females **(A)**. Photomicrographs of GHRH immunoreactivity staining **(B)** and GHRH positive cells **(C)** in the hypothalamus of 36-week-old male and female *Ghrl+/+* and *Ghrl-/-* mice. Data represent mean ± SEM. Number of mice in panel **(A)** males (n=4 *Ghrl+/+* males and 4 *Ghrl-/-*), females (n=4 *Ghrl+/+* and 4 *Ghrl-/-*). Number of mice in panel **(C)** males (n=3 *Ghrl+/+* males and 5 *Ghrl-/-*), females (n=5 *Ghrl+/+* and 8 *Ghrl-/-*) *p < 0.05 *Ghrl-/- versus Ghrl+/+* mice of the same sex, ^&&^p < 0.01, ^&&&^p <0 .0001 KCl *versus* base and GHRH.

To test if the alteration of the ultradian GH pattern in adult *Ghrl*-/- females was due to modifications at the pituitary level, we assessed basal and stimulated GH secretion from pituitary gland explants (**Figure 3A**). As reported earlier (26), basal and GHRH-induced GH release were significantly higher in males than in females (ANOVA, sex effect: p<0.0001). However, basal GH release or GH release stimulated by GHRH or KCl was not different in pituitary explants from *Ghrl+/+* and *Ghrl-/-* mice in either sex.

### The Number of GHRH+ Neurons Increased in ArcN of Adult Female, but Not Male, *Ghrl-/-* Mice

The hypophysiotropic GHRH neurons of the ArcN control the pulsatile pattern of GH secretion (39) and are sexually dimorphic (47). Thus, we estimated the sex and genotype effects on GHRH immunoreactive signal in the ArcN. Our analysis measuring the visualized GHRH+ cells in the ArcN revealed a significant interaction between sex and genotype (ANOVA, sex effect: p=0.7901, genotype effect: p=0.1185, sex x genotype interaction: p=0.0145) (**Figures 3B, C**). The number of visualized GHRH+ neurons increased in *Ghrl*-/- females, as compared to *Ghrl*+/+ females (p<0.05, *post-hoc* test), whereas the number of GHRH+ neurons of *Ghrl-/-* and *Ghrl+/+* males was not different. In the hypothalamus, no genotype differences were found in the mRNA levels of *Ghrh* (1.00 ± 0.14 in *Ghrl+/+* males, 1.18 ± 0.14 in *Ghrl-/-* males, 0.74 ± 0.10 in *Ghrl+/+* females, 0.82 ± 0.19 in *Ghrl-/-* females) (ANOVA, sex effect: p=0.0599, genotype effect: p=0.3961, sex x genotype interaction: p=0.7589), or *Ghsr* (1.00 ± 0.08 in *Ghrl+/+* males, 0.92 ± 0.10 in *Ghrl-/-* males, 0.83 ± 0.07 in *Ghrl+/+* females, 0.69 ± 0.02 in *Ghrl-/-* females) (ANOVA, sex effect: p=0.0149, genotype effect: p=0.1482, sex x genotype interaction: p=0.6274; with p<0.05 only for *Ghrl-/-* females *vs*. *Ghrl+/+* males, *post-hoc* test).

## Discussion

As compared to wild-type littermates, *Ghrl-/-* mice of both sexes lack major alterations in body growth, metabolic parameters or feeding behavior at adulthood. Adult *Ghrl-/-* females display, however, decreased amplitude of GH secretion and increased irregularity of secretion accompanied by increased immunoreactive signal for GHRH in the ArcN without change in the pituitary GH content or GH secretion from pituitary explants.

### Ghrl Deletion Affects Pulsatile GH Secretion and Regularity of Ultradian GH Pattern in Adult Female, but Not Male, Mice

Among the proghrelin-derived peptides, ghrelin seems to play the most powerful control on GH secretion. Indeed, ghrelin treatment potently increases GH secretion in humans and rodents. However, the role of endogenous ghrelin on GH secretion has been more difficult to demonstrate. Endogenous ghrelin seems to regulate the amplitude of GH secretion in healthy men since ghrelin levels and GH pulse amplitude strongly correlate (49). Pharmacological blockage of GHSR in male rats decreased the amplitude of GH release but did not affect the pattern of GH secretion (50). Also, no correlation was found between acyl ghrelin or total ghrelin and GH plasma peaks in freely behaving male rats (1, 51). In male rats, we previously showed that ghrelin displays an ultradian pattern of secretion through the light and dark periods with pulses occurring with a frequency similar to GH (1). In mice, however, due to the small volume of blood and the absence of ghrelin assays with sufficient sensitivity, determining the ultradian pattern of ghrelin secretion and a possible sexual dimorphic pattern of secretion are challenging. To our knowledge, few studies have investigated the link between endogenous ghrelin and GH secretion in women or female rodents. Transgenic female, not male, rats with reduced hypothalamic GHSR expression display reduced pulsatile GH secretion (52). In contrast to adult males, adult *Ghsr*-/- female mice showed a reduction of total, basal and pulsatile GH secretion (37). Furthermore, *Ghrl*-/- and *Goat-/-* male mice lack alterations of pulsatile GH secretion at adulthood, although some transitory modifications are seen in young 7-8-week-old males, a period of rapid growth rate (26, 53), suggesting that ghrelin-gene derived peptides regulate pulsatile GH secretion in an age-dependent manner.

Here, we observed that adult *Ghrl*-/- males lack significant alterations in GH secretion whereas adult *Ghrl*-/- females display a reduction of pulsatile GH secretion, maximal pulse amplitude and regularity of GH secretory pattern (*i.e.*, higher ApEn values). ApEn is considered a barometer of GH negative feedback mechanism on GH release. As observed in all species investigated so far (39), we confirmed that females show higher ApEn values and reduced pulsatile GH secretion as compared to males. The increased ApEn values in *Ghrl*-/- females are in line with the reduced pulsatile GH secretion, whereas the unchanged ApEn values in *Ghrl*-/- males point to unchanged pulsatile GH secretion, as observed herein. Of note, the higher interindividual variability observed in males for several parameters of GH secretion may have prevented from unmasking statistical genotype differences in this sex. In any case, our data demonstrate a significant effect of endogenous proghrelin-derived peptides on GH secretion in adult female mice. Since both *Ghsr*-/- females and *Ghrl*-/- females show a reduction of the pulsatile GH secretion, it seems reasonable to hypothesize that endogenous ghrelin-evoked GHSR signaling increases GH secretion in adult female mice mainly. It is important to highlight that stress is known to reduce plasma GH levels (54). The blood sampling procedure used in the current study induces only a modest rise in plasma corticosterone levels that are within the physiological range of endogenous diurnal variations (53). Furthermore, *Ghrl-/-* mice displayed similar plasma corticosterone levels than *Ghrl+/+* mice in non-stressed conditions and in response to restrain-stress (unpublished observations). Thus, it seems unlikely that differential responses to the experimental blood sampling procedure may have contributed to reduce GH secretion in *Ghrl-/-* females.

### Neuroendocrine Basis by Which Ghrl Deletion Affects GH Pulse Amplitude and Regularity of Ultradian GH Pattern in Female Mice

Ghrelin-induced GH secretion involves pituitary and hypothalamic mechanisms (39). *In vitro*, ghrelin acts on somatotropic cells of the pituitary to release GH (55). Also, ghrelin enhances GHRH-induced GH release and impairs somatostatin-mediated inhibition of GH release. Notably, ghrelin treatment does not induce GH secretion in male mice lacking GHSR exclusively in somatotropic cells (56). Ghrelin also indirectly acts at the pituitary by stimulating GHRH neurons and inhibiting somatostatin neurons *via* multiple mechanisms. For instance, ghrelin directly excites GHRH neurons, and also indirectly increases GHRH neuron excitability by decreasing their inhibitory GABA inputs (57). Here, we found that *Ghrl-/-* and *Ghrl+/+* females show similar pituitary GH contents as well as a similar basal, KCl-induced and GHRH-induced secretion of GH *in vitro*. Conversely, a higher number of GHRH+ neurons was visualized in the ArcN of *Ghrl-/-* females, as compared to *Ghrl+/+* females, and no changes in the GHRH levels in the median eminence (not shown) nor in the hypothalamic levels of *Ghrh* mRNA. Thus, hypothalamic, rather than pituitary dysfunctions are more likely associated to the reduction of the pulsatile GH secretion in *Ghrl-/-* females. Transgenic female rats with reduced GHSR expression in hypothalamic tyrosine hydroxylase-expressing neurons show reduced GH secretion and lower number of GHRH neurons (58), also suggesting hypothalamic basis for altered GH secretion induced by a deficit of ghrelin action in female rodents. Importantly, the GHRH neurons are poorly visualized in naive rodents because the GHRH peptide is rapidly transported to the neuron terminals for release (59). Thus, the above referred study in rats used colchicine treatment to estimate the number of GHRH neurons. Here, however, the GHRH immunolabeling was performed in brain sections of naive mice undermining our capacity to estimate the number of GHSR neurons in each experimental group. Since *Ghrl-/-* females display a reduction of pulsatile GH secretion, it seems reasonable to hypothesize that GHRH neurons were more easily visualized in *Ghrl-/-* females because GHRH secretion was reduced. Such hypothesis could be further tested by assessing plasma GH levels in *Ghrl+/+* and *Ghrl-/-* females following somatostatin withdrawal, which triggers GHRH-dependent GH secretion *in vivo* (60). In this regard, it is possible that somatostatin neurons, which control the timing of GH secretory pulses and whose release activity is also under the control of ghrelin (4), are differentially affected in *Ghrl-/-* females and contribute to modulate GH secretion. Of note, expression of somatostatin receptor 2 on GHRH neurons displays sexual dimorphism in mice, suggesting that somatostatin interacts with GHRH neurons to control sexual dimorphic GH responses (47). Further studies are required to precisely determine the hypothalamic dysfunctions affecting the GH secretion in *Ghrl-/-* females. Also, the reason why alterations of GH secretion only take place in females remain uncertain. Of note, the ghrelin system displays important dimorphic responses (34). Indeed, studies in women showed that estradiol potentiates ghrelin-stimulated pulsatile GH secretion and GHRH/ghrelin synergy (61–63). In mice, females were also more sensitive to the GH-releasing effects of ghrelin treatment than males (34). Strikingly, we found that Ghsr mRNA levels in the hypothalamus were not different among genotypes, although Ghsr mRNA levels tended to be lower in *Ghrl-/-* than *Ghrl+/+* females. Previously, Ghsr mRNA levels were found similar in the ArcN of 8-week-old male and female rats (64) and slightly lower in the hypothalamus of 6-month-old female rats, as compared to males (65).

### Ghrl Deletion in Mice Does Not Significantly Affect Food Intake, Growth or Metabolism at Adulthood

As previously reported (12, 23, 25), we confirmed that adult *Ghrl-/-* mice – male and female - lack alterations of spontaneous food intake, meal pattern or fast-refeeding hyperphagia. Also, adult *Ghrl-/-* mice show no major growth or metabolic deficits, as previously shown (26), despite adult *Ghrl-/-* females show alterations of their GH secretion pattern. Notably, previous studies also showed that young *Ghrl-/-* males display alterations in ultradian GH secretion patterns that are not associated with changes in growth or metabolic parameters (26). Similarly, young *Goat-/-* mice show reduced GH pulse amplitude but unaltered body weight or body growth, although an increase in plasma insulin-like growth factor I levels has been proposed to compensate the alterations in GH secretion in these mice (53). Thus, the lack of proghrelin-derived peptides seems to induce some sexually dimorphic alterations in the GH axis that are insufficient to affect eating behaviors, body growth and metabolism at adulthood. Still, altered GH secretion during adulthood could contribute to specific phenotypes associated with ageing or pathophysiological conditions. Indeed, we observed a tendency to reduced volume of mineralized bone and significant reductions in the mass of soleus muscle in older 80-week-old *Ghrl-/-* mice that may be linked to ghrelin since this hormone plays a protective effect on bones structure in older mice (66) and both acyl and des-acyl ghrelin enhance muscle anabolism (67) and prevent skeletal muscle atrophy (68).

### The Absence of Evident Dysfunctions in Adult Mice Lacking Ghrl Gene Must Be Interpreted With Caution

The study of mice with genetic modifications of different elements of the ghrelin system, such as proghrelin, GOAT or GHSR, has been instrumental to reveal some aspects of their physiological role. For instance, the observation that *Goat-/-* mice, in contrast to WT mice, suffer severe hypoglycemia and become moribund in a starvation protocol indicates that ghrelin plays essential functions under energy deficit (21). In this regard, it is interesting to stress that *Ghrl-/-* mice exhibit less evident alteration than mice lacking GHSR [as review in (12)]. For instance, GHSR-deficient mice, but not *Ghrl*-/- mice, show reduced hyperphagia after fasting (25). GHSR-deficient mice show some alterations, as compared to *Ghrl-/-* mice, even under *ad libitum* feeding conditions. In contrast to *Ghrl-/-* mice, adult *Ghsr*-/- mice fed on a regular chow displayed reduced body weight and linear growth, regardless of the sex (37). Furthermore, meal frequency was reduced in *Ghsr*-/- male mice (37). The exact molecular mechanisms underlying such distinct phenotypes in *Ghsr*-/- mice, as compared to *Ghrl-/-* mice, are uncertain. On one hand, GHSR acts *via* several ligand-independent mechanisms that include its capacity to induce constitutive intracellular signaling or its ability to cross-talk with other receptors (69). Also, the deletion of *Ghrl* gene not only eliminates ghrelin but also obestatin and des-acyl-ghrelin, which seem to impair the effect of ghrelin. For instance, obestatin treatment antagonizes ghrelin-induced GH secretion in rats (70), although these observations were not confirmed by others (71, 72). Des-acyl ghrelin appears to block some of the effects of ghrelin *via* either GHSR-dependent or GHSR-independent mechanisms (12, 73, 74). Finally, the liver-expressed antimicrobial peptide 2 (LEAP2) was recently recognized as another endogenous peptide ligand for GHSR (75). LEAP2, which is released by endocrine cells of the liver and the intestinal tract, abrogates ghrelin-evoked and constitutive GHSR activities (75, 76). Notably, LEAP2 displays a binding affinity to GHSR similar to ghrelin, but its level in plasma is ~10-fold higher than ghrelin level in *ad libitum* fed conditions (69). Thus, LEAP2 may play a more important role than ghrelin on GHSR under the tested conditions, masking some of the consequences of *Ghrl* gene deletion.

In conclusion, present data suggest that ghrelin plays a more prominent role in the regulation of pulsatile GH secretion in adult female than male mice. The mechanism of the sex-specific effect of preproghrelin deletion on GH pulsatility still needs to be refined. The physiological implications of the altered GH pulsatility in preproghrelin deleted mice would also need to be clarified since it is not associated with major growth, feeding or metabolic phenotypes.

## Data Availability Statement

The raw data supporting the conclusions of this article will be made available by the authors, without undue reservation.

## Ethics Statement

The animal study was reviewed and approved by Animal Experimentation Committee CEEA.34 of University of Paris.

## Author Contributions

RH, GF, MP, JE, and VT designed experiments. RH, GF, NL, AL, OF, PZ, and OV performed experiments. FR performed jkApEn and deconvolution analysis of GH pulsatility parameters. CC performed all studies and analysis on bone parameters. CT provided the colony of preproghrelin deficient mice. RH, GF, NL, MP, and VT analyzed data. MP and VT wrote the manuscript. JE edited the manuscript. All authors contributed to the article and approved the submitted version.

## Funding

This work was supported by an Agence Nationale de la Recherche Jeunes Chercheuses Jeunes Chercheurs Grant ANR-12-JSV1–0013-01 (to VT), Institut National de la Santé et de la Recherche Médicale (INSERM), grants from the Fondo para la Investigación Científica y Tecnológica (FONCyT, PICT2016-1084 and PICT2017-3196) and from CONICET (PUE-2017) to MP. GF was supported by CONICET.

## Conflict of Interest

The authors declare that the research was conducted in the absence of any commercial or financial relationships that could be construed as a potential conflict of interest.

## Publisher’s Note

All claims expressed in this article are solely those of the authors and do not necessarily represent those of their affiliated organizations, or those of the publisher, the editors and the reviewers. Any product that may be evaluated in this article, or claim that may be made by its manufacturer, is not guaranteed or endorsed by the publisher.
