# Supplementary material for: Ghrelin Gene Deletion Alters Pulsatile Growth Hormone Secretion in Adult Female Mice
Source: Front Endocrinol (Lausanne). 2021 Oct 13;12:754522. doi: 10.3389/fendo.2021.754522 (PMC8549963; doi:10.3389/fendo.2021.754522)
Supplement: Supplementary file 1 [file DataSheet_1.docx]

Supplementary Material

1. **Exploration of pulsatile GH secretion.**
   1. **Repeated blood sampling for pulsatile GH explorations**.

Mice were habituated to handling and blood sample collection by performing daily a gentle massage of the tail 1 week before the beginning of the experiment to minimize stress. Whole blood samples were collected as previously described [1], except that the tail was slightly incised 1 hour prior to blood collection and no cardboard tube was used to handle the mouse during sampling. Blood samples (2 μL) were collected and homogenized with 58 μL of the enzyme immunoassay (EIA) GH buffer (PBS 1X-0.05% Tween 20) (whole blood dilution 1:30) on 96-well plates kept on ice during the whole procedure and then stored at -20°C until assayed.

- 1. **GH enzyme immuno-assay on whole blood samples.**

Whole-blood GH concentrations were evaluated by EIA as previously described [1] except that values were reported in terms of rat GH-RP2 (see below for detailed protocol). The assay sensitivity was 0.038 ng/mL, and intra- and inter-assay coefficients of variations were 3.2% and less than 8.75%, respectively. Values were expressed as plasma GH concentrations. In details, a 96-well plate was coated with 50 μl capture antibody (NIDDK-anti-rGH-IC-1 8 (Monkey), AFP411S, NIDDK-NHPP, at a final dilution of 1:40 000) and incubated overnight at 4°C. To reduce non-specific binding, each well was subsequently incubated with 200 μl blocking buffer: 5% skim milk powder in 0.05% phosphate buffered saline with Tween (0.05% PBS-T). A standard curve (0.03 to 8 ng/mL) was generated using a two-fold serial dilution of rat GH (rGH-RP2, AFP 3190B, NIDDK NHPP) in PBS-T. Bound standards and samples (whole blood diluted 1:50 in PBS-T) were incubated with 50 μl detection antibody (rabbit antiserum to rGH, AFP5672099, NIDDK-NHPP, at a final dilution of 1:40 000 in blocking buffer). The bound complex was incubated with 50 μl horseradish peroxidase-conjugated antibody (Polyclonal Goat Anti-Rabbit IgG/HRP, P0448, DakoCytomation, Agilent, Santa Clara, CA,USA) at a final dilution of 1:2000 in 50% blocking buffer and 50% 1X PBS. Addition of 100 μl O-phenylenediamine (00-2003, Invitrogen, Carlsbad, CA) substrate dissolved in Citrate Buffer to each well resulted in an enzymatic colorimetric reaction. This reaction was stopped by addition of 50 μl 3M HCl and the absorbance was read at a wavelength of 490 nm with a microplate reader (Multiskan EX SN ThermoFisher). The concentration of GH in each well was calculated using a 4-parameters non-linear regression of the standard curve. GH values were then multiplied by the factor of recovery of GH in whole blood compared to plasma, i.e. 1.72 (100/58.25) (as previously reported in [1], there is a 58.25 ± 5.57% recovery of GH in whole blood samples when compared with plasma) in order to present data as GH plasma concentrations.

- 1. **Deconvolution analyses of GH secretion profiles.**

The GH concentration time series were analyzed using an automated multiparameter deconvolution method, which was empirically validated using hypothalamo-pituitary sampling and simulated pulsatile time series [2; 3]. Sensitivity and specificity both exceed 93%. Deconvolution essentially transforms a hormone concentration time series in secretory events (pulse mass and time of onset), non-pulsatile secretion (basal secretion), hormone half-lives (usually the fast component is a fixed value) according to a particular *a priori* model of hormone secretion and clearance and the pulse secretion form (generally not a Gaussian form). The individual pulses (mass) identified in each animal were not used in this manuscript, only the integrated sum, which is the pulsatile secretory component, and the mean pulse mass (MPM) for each animal. Secretion not explained by pulsatile secretion is defined as basal secretion, and is calculated by the automated program.

The Matlab-based algorithm first detrends the data and normalizes concentrations to the unit interval [0, 1]. Second, the program creates multiple successively decremental potential pulse-onset time sets (by locating the sign change of the first derivative), each containing one fewer pulse by a smoothing process (a nonlinear adaptation of the heat-diffusion equation). Third, a maximum-likelihood (MLE) estimation (MLE) method calculates all secretion and elimination parameters simultaneously conditional on each of the candidate pulse-time sets. Deconvolution parameters comprise basal secretion (β_0_), two half-lives (α_1_, α_2_), secretory burst mass (η_0_, η_1_), random effects on burst mass (σ_A_), procedural/measurement error (σ_ε_), and a three-parameter flexible gamma secretory-burst waveform (γ_1_, γ_2_, γ_3_). The fast half-life was represented here as 1 min, constituting 37% of the decay amplitude and the slow half-life as an unknown variable. Statistical model selection was performed to distinguish among independently framed fits of the multiple candidate pulse-time sets using the Akaike information criterion with a pulse number penalty. The parameters (and units) of interest here are the slow half-life (minutes), basal (i.e. non-pulsatile secretion) and pulsatile secretion (sum the secretion by the individual identified statistically significant pulses expressed as mass units/time interval, here ng/mL/6 hours), and waveform shape (mode [the time delay to maximal secretion after objectively estimated pulse onset], minutes). The program requires active input of initial estimates of the parameters, e.g. slow half-life range 5 -11 min, slow-wave amplitude fraction 0.63, mode range 3-12 min, number of pulses in 6 hours, range 2-9 pulses, and initial waveform parameter vector [2; 5; 10] (dimensionless). An in-depth review of the methods for analyzing pulsatile hormone secretion is available for the non-mathematically trained reader [4].

Approximate Entropy (ApEn) is a scale- and model-independent univariate regularity statistic used to quantify the orderliness (subpattern consistency) of serial stationary (nontrending) measurements [5]. Higher ApEn defines reduced regularity of hormone secretion, which in general typifies puberty, aging, diminished negative feedback due to target-gland failure, fixed exogenous stimulation, and autonomous neuroendocrine tumors [6]. The statistic is calculated on a time series as a single, finite, positive, real number JkApEn which is the jackknived version of the ApEn algorithm.

1. **GHRH immunostaining**

Chromogenic immunohistochemistry against GHRH was performed using a previously validated anti-GHRH antiserum raised in rabbit against the 25-residue C-terminal end of the mouse GHRH sequence conjugated to keyhole limpet hemocyanin as carrier [7]. The antiserum does not cross-react with rat GHRH, human GHRH, somatostatin, TRH or cortistatin. Importantly, anti-GHRH immunolabeling is completely abolished by preincubation with mouse GHRH, and coincides with the localization of the eGFP neurons in colchicine-treated GHRH-eGFP mice, in which eGFP expression is under the control of the GHRH promote [7]. For immunostaining, sections were pre-treated with 0.5% H2O2, blocking solution (2% normal horse serum and 0.25% Triton-X) and incubated with a rabbit anti-GHRH antibody (immunoserum L0851, 1:10000) for 48-h at 4°C. Next, sections were treated with a biotinylated donkey anti-rabbit antibody (1:4000, Jackson Laboratories, cat# 711-065-152) for 1 h, and with the Vectastain Elite ABC kit (Vector Laboratories, cat# PK-6200) for 1-h. Finally, visible black/purple signal was developed with diaminobenzidine/nickel solution. Brain sections were mounted on glass slides and coverslipped with mounting media. Low- and high-magnification bright-field images were acquired with 10X/0.30 and 40X/0.65 objectives using a Nikon Eclipse 50i and a DS-Ri1 Nikon digital camera. All images were taken in comparable areas and under the same optical and light conditions.

1. **Assessment of spontaneous food intake in automated cages**

Mice were habituated to single housing into the automated drinking/feeding station (LabMaster, TSE systems, GmbH, Germany) for 1 week before the beginning of the experiment. The first three days of recording were considered as the habituation period. Test cages were equipped with food and drink high precision sensors of 10 mg and 10 µl resolution, respectively. Mice had free access to food and water during the whole recording period. A meal consisted of the consumption of at least 30 mg of food separated from the next feeding episode by at least 10 min. For each mouse, inter-meal interval (time between the end of a meal and the beginning of the next one), meal duration, meal size (weight of food consumed in a meal), meal number, meal rate, were assessed as previously reported [8].

1. **Assessment of tibia microstructure**

Tibias from a cohort of mice (6-8 Ghrl+/+ and 8 Ghrl-/- males; 7-8 Ghrl+/+ and 4-5 Ghrl-/- females) were dissected and cleaned of connective tissue, and triceps surae muscle (including soleus and both lateral and medial heads of the gastrocnemius) was weighed. After an overnight fixation in 4% paraformaldehyde at 4°C, tibias were transferred in 70% ethanol and kept at 4°C until micro-computed tomography (CT) analyses. Tibias were scanned using micro-computed tomography (Bruker Skyscan 1172, Kontich, Belgium). The X-ray source was set at voltage of 80 kV and a current of 100 µA. A pixel size of 10 µm, an integration time of 2350 ms, a rotation step of 0.7, an averaging frame of 2, and an aluminum/copper filter were used. Acquired images were reconstructed using NRecon software (Bruker, Kontich, Belgium). All images were compensated for misalignments and the adjustment of the reconstruction parameters was as follows: beam hardening factor correction = 25%, ring artefact reduction = 6 and smoothing = 2. Images were reoriented using Dataviewer software (Bruker) and tibia length measured using CTAn software (Bruker, Kontich, Belgium). The following parameters of trabecular bones were analyzed: volume of mineralized bone per unit volume of the sample (Bone volume fraction, BV/TV), Number of trabecules (Trabecular number, Tb.N.), Mean thickness of the trabeculae (Trabecular thickness, Tb.Th.), Mean distance between the trabeculae (Trabecular separation, Tb.Sp.) and Structure Model Index (SMI).

1. **GH pituitary content and GH release from pituitary explants *ex vivo***

For measurement of GH content, pituitaries were dissected and homogenized by sonication in 200 µl NaHCO_3_ 0.1M. After centrifugation, supernatants were frozen at -20°C until GH content determination. Total protein contents were determined by the Peterson method [9].

For measurement of basal and GHRH-stimulated GH release *ex vivo*, pituitaries were dissected, placed in perifusion chambers and superfused at a rate of 0.1 ml/min with oxygenated Dulbecco’s modified Eagle’s medium containing bovine serum albumin (0.1%), NaHCO_3_ (1 g/L), glutamine (2 mM) and glucose (4.5 g/L). After a 120 min equilibration period, effluents were collected every 5 minutes. Pituitaries were first perifused with medium alone to determine basal GH release then with medium containing GHRH (10^-7^M) (Phoenix Peptide) to determine GHRH-stimulated GH release, then with medium alone. Finally, KCl (25 mM) was added to the medium to verify the explants viability. Samples were frozen at -20°C until GH determination.

1. **Real-time quantitative PCR measurement of hypothalamic GHRH and GHSR mRNA levels**

Expression of hypothalamic GHRH (Mm00439098-m1) and GHS-R (Mm00616415_m1) was quantified using TaqMan gene expression assays (Applied Biosystems Inc., Foster City, CA, USA). *Cyclophilin A* (Mm02342430_g1) and GAPDH (Mm99999915_g1) were used as housekeeping genes. Relative quantification (RQ) was calculated as the fold change in gene expression normalized to the mean of the two housekeeping genes and expressed relative to the male *Ghrl*+/+ group.

**References**

[1] F.J. Steyn, L. Huang, S.T. Ngo, J.W. Leong, H.Y. Tan, T.Y. Xie, A.F. Parlow, J.D. Veldhuis, M.J. Waters, and C. Chen, Development of a method for the determination of pulsatile growth hormone secretion in mice. Endocrinology 152 (2011) 3165-71.

[2] D.M. Keenan, F. Roelfsema, N. Biermasz, and J.D. Veldhuis, Physiological control of pituitary hormone secretory-burst mass, frequency, and waveform: a statistical formulation and analysis. American journal of physiology. Regulatory, integrative and comparative physiology 285 (2003) R664-73.

[3] P.Y. Liu, D.M. Keenan, P. Kok, V. Padmanabhan, K.T. O'Byrne, and J.D. Veldhuis, Sensitivity and specificity of pulse detection using a new deconvolution method. Am J Physiol Endocrinol Metab 297 (2009) E538-44.

[4] J.D. Veldhuis, D.M. Keenan, and S.M. Pincus, Motivations and methods for analyzing pulsatile hormone secretion. Endocrine reviews 29 (2008) 823-64.

[5] G. Meyfroidt, D.M. Keenan, X. Wang, P.J. Wouters, J.D. Veldhuis, and G. Van den Berghe, Dynamic characteristics of blood glucose time series during the course of critical illness: effects of intensive insulin therapy and relative association with mortality. Crit Care Med 38 (2010) 1021-9.

[6] J.D. Veldhuis, M.L. Johnson, O.L. Veldhuis, M. Straume, and S.M. Pincus, Impact of pulsatility on the ensemble orderliness (approximate entropy) of neurohormone secretion. American journal of physiology. Regulatory, integrative and comparative physiology 281 (2001) R1975-85.

[7] K. Bouyer, C. Loudes, I.C. Robinson, J. Epelbaum, and A. Faivre-Bauman, Sexually dimorphic distribution of sst2A somatostatin receptors on growth hormone-releasing hormone neurons in mice. Endocrinology 147 (2006) 2670-4.

[8] R. Hassouna, P. Zizzari, O. Viltart, S.K. Yang, R. Gardette, C. Videau, E. Badoer, J. Epelbaum, and V. Tolle, A natural variant of obestatin, Q90L, inhibits ghrelin's action on food intake and GH secretion and targets NPY and GHRH neurons in mice. PLoS One 7 (2012) e51135.

[9] G.L. Peterson, A simplification of the protein assay method of Lowry et al. which is more generally applicable. Analytical biochemistry 83 (1977) 346-56.
